# Local and regional anaesthesia in dogs and cats: Overview of concepts and drugs (Part 1)

**DOI:** 10.1002/vms3.219

**Published:** 2020-01-21

**Authors:** Tamara Grubb, Heidi Lobprise

**Affiliations:** ^1^ Washington State University Pullman WA USA; ^2^ Main Street Veterinary Hospital Flower Mound TX USA

**Keywords:** analgesia, bupivacaine, liposome‐encapsulated, local anaesthetics, local block

## Abstract

Pain management in veterinary patients is a crucial component of appropriate patient care. Multimodal analgesia that includes both systemically and locally/regionally administered drugs is generally the most effective approach to providing pain relief. Local anaesthetic drugs used in local and regional blockade are unique in that they can completely block the transmission of pain (in conscious patients) or nociceptive (in anaesthetized patients) signals, thereby providing profound analgesia. In addition, local and regional administration of drugs, when compared with systemic bolus administration, generally results in a lower incidence of dose‐related adverse effects. Due to the potential to provide profound analgesia and the high safety margin (when used correctly) of this drug class, local anaesthetics are recommended as part of the analgesic protocol in the majority of patients undergoing surgical procedures or suffering traumatic injuries. This manuscript, Part 1 of a two‐part instalment, emphasizes the importance of using local and regional anaesthesia as a component of multimodal analgesia, provides a review of the basic pharmacokinetics/pharmacodynamics of local anaesthetic drugs in general, lists information on commonly used local anaesthetic drugs for local and regional blockade in dogs and cats, and briefly introduces the novel liposome‐encapsulated bupivacaine (NOCITA®). Part 2 is a review of local and regional anaesthetic techniques used in dogs and cats (Grubb & Lobprise, 2020).

## Graphical abstract

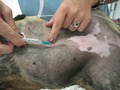

## 1 INTRODUCTION

Provision of effective analgesia is a crucial component of appropriate care for patients experiencing pain, including acute surgical and traumatic pain (Lascelles & Kirkby‐Shaw, 2016). Note that ‘pain’ is used to describe the sensation in conscious patients but the sensation is referred to as ‘nociception’ in anaesthetized patients since a cognitive response, which is prevented by the anaesthetic, is necessary to define pain. Acute pain can be treated by a variety of drug classes including opioids, anti‐inflammatory drugs and local anaesthetic drugs. Local anaesthetic drugs are unique in that their analgesic effects are produced following local or regional, rather than systemic (i.e. IV, IM, SQ, PO), administration. This results in decreased likelihood of adverse effects that might be caused by systemic bolus administration of drugs. Lidocaine is the only local anaesthetic that can be administered systemically, but the focus of our manuscripts is the local and regional administration.

Local anaesthetic drugs are also unique in that, unlike drugs such as opioids that *modulate* pain (or nociceptive) impulses once they reach the central nervous system (CNS), local anaesthetics *prevent* the pain (or nociceptive) impulse from reaching the CNS, which is a very specific and powerful role in the nociceptive pathway. Local anaesthetics primarily block sodium channels in nerves, which prevents nerve depolarization and propagation of the action potential, thus preventing propagation of the pain stimulus. Blockade of pain signals may have a more profound impact than modulation of pain signals as humans receiving local blocks alone had lower pain scores and a reduced requirement for rescue analgesia than those receiving systemic opioids alone following thoracic limb surgery (Rodríguez et al., 2018). In veterinary medicine, dogs receiving local blocks following a thoracotomy had lower pain scores than those receiving systemic opioids (Conzemius, Brockman, King, & Perkowski, 1994).

However, each of the analgesic drug classes previously listed provides analgesia via different mechanisms of action, thus each is an appropriate and important component of multimodal analgesic protocols. When used as part of a multimodal protocol, both intraoperative nociceptive indicators (e.g. heart rate, respiratory rate and blood pressure changes at the time of a nociceptive stimulus) and postoperative pain scores are lower in patients receiving local/regional anaesthesia along with systemically administered analgesics when compared to patients receiving systemically administered analgesics alone (Aguiar, Chebroux, Martinez‐Taboada, & Leece, 2015; Benito et al., 2016; Carpenter, Wilson, & Evans, 2004; Mosing, Reich, & Moens, 2010; Myrna, Bentley, & Smith, 2010; Perez et al., 2013; Savvas et al., 2008; Yilmaz et al., 2014).

Another advantage of intraoperative anti‐nociception is improved anaesthetic safety because the inhalant dose, or minimum alveolar concentration (MAC), required to produce a surgical plane of anaesthesia is decreased in patients receiving local/regional blocks as part of a multimodal analgesic protocol (Aguiar et al., 2015; Kona‐Boun, Cuvelliez, & Troncy, 2006; McMillan, Seymour, & Brearley, 2012; Mosing et al., 2010; Perez et al., 2013; Snyder & Snyder, 2013). Decreased inhalant dosages result in a reduction in the dose‐dependent cardiorespiratory effects of the inhalants, thereby promoting improved anaesthetic safety (Snyder & Snyder, 2013). Decreased inhalant dosages secondary to local/regional blockade may also play a role in increased survival from cancer, as inhalants appear to suppress cell‐mediated immunity and allow proliferation of tumour cells (Kim, 2017).

In addition to decreased inhalant dosing, the need for rescue analgesia, including opioids, is decreased with the use of local/regional analgesia. In humans (Bergese et al., 2012; Blanco, Ansari, & Girgis, 2015; Boerboom et al., 2018; Candiotti, 2012; Lombardi, 2014; Malik, Kaye, Belani, & Urman, 2017; Marques et al., 2014; Stokes et al., 2017) and dogs/ cats (Benito et al., 2016; Carpenter et al., 2004; Flecknell, Kirk, Liles, Hayes, & Dark, 1991; Myrna et al., 2010; Perez et al., 2013; Savvas et al., 2008; Wenger, Moens, Jäggin, & Schatzmann, 2005), local anaesthetics significantly decrease the opioid requirements for intraoperative anti‐nociception and postoperative analgesia. Using local anaesthetics to decrease opioid use could be very beneficial in both human and veterinary patients, not only because of the desire to reduce potential opioid‐mediated adverse effects, such as dysphoria, vomiting and nausea, but also because the availability of potent opioids may be limited due to legislation, production or country/region.

Finally, in addition to provision of intraoperative anti‐nociception and immediate postoperative analgesia, local anaesthetics may decrease the incidence of intermediate duration (i.e. several days or a few weeks) and chronic (i.e. months to years to end of life) pain. The intensity and duration of pain in recovery is an important indicator of the likelihood of chronic pain development in humans (Althaus et al., 2018; Boerboom et al., 2018; de Brito, Omanis, Ashmawi, & Torres, 2012; Jin et al., 2016; Rashiq & Dick, 2014; Voscopoulos & Lema, 2010). The fact that human patients with local anaesthetic drugs included in the analgesic protocols were less likely to develop both postoperative and chronic pain is a very compelling reason to utilize local/regional blockade (Boerboom et al., 2018; de Brito et al., 2012; Rashiq & Dick, 2014). Although no studies are available for animals, the authors suggest that, based on the similarity of the mammalian pain pathway across species, the same result could be extrapolated for veterinary patients, including dogs and cats. Due to the fact that both intensity and duration of acute pain may promote chronic pain development, more profound and longer lasting analgesics and/or analgesic techniques should play a prominent role in postoperative analgesia. Thus, utilizing local anaesthetics administered through wound diffusion catheters or administering liposome‐encapsulated bupivacaine, which provides postoperative pain relief of up to 72‐hr in the dog (Lascelles, Rausch‐Derra, Wofford, & Huebner, 2016) and cat (NOCITA® product insert website), may be a beneficial addition to postoperative multimodal protocols.

Due to their ability to profoundly decrease both intraoperative nociception and postoperative pain, local anaesthetic drugs are recommended for use in the majority of surgical procedures and traumatic injuries, as outlined in recent veterinary pain management guidelines (Epstein et al., 2015; Mathews et al., 2014).

### 1.1 Local anaesthetic drug properties

The general information on the mechanism of action of local anaesthetics, nerve fibre types and their specific function, along with time to onset and duration of action of different local anaesthetics, is commonly published and can be referenced from many sources (Berde & Strichartz, 2000; Catterall & Mackie, 2001; Scholz, Salinas, Spencer, & Liu., 2004). Veterinary‐focused reviews have been published (Campoy & Read, 2013; Rioja Garcia, 2015). Original research is not available for much of this information but is included where possible. Specific information in dogs and cats is included where possible.

As stated, local anaesthetics block sodium channels in the nerve to block propagation and transmission of nociceptive impulses. The presence of myelination, nerve fibre diameter and firing frequency impact the order and likelihood of blockade and contribute to the ‘selective’ or ‘differential’ blockade of the nerves, which was first described in 1929 (Gasser & Erlanger, 1929). Nerves are divided into groups (A, B, C) according to size and myelination. Group A fibres are large, myelinated fibres that transmit signals associated with motor function of muscles (A‐α); touch, pressure and proprioception (A‐β); and intense, early onset (or ‘fast’) pain (A‐δ). Group B are small, myelinated fibres primarily associated with autonomic function like vasomotor control. Group C are small, unmyelinated fibres that transmit signals associated with temperature and low‐level or ‘dull’ pain. Although not entirely straight forward, *in general*, following the administration of local anaesthetics, the fibres in Group B are desensitized first, followed by the C‐ and A‐δ fibres, then by the A‐ β fibres (Rioja Garcia, 2015). The larger‐diameter fibres in Group A are the most blockade resistant, thus motor function is the last to be blocked or may not be blocked at all (Rioja Garcia, 2015). Return to normal signal transmission appears to occur in the opposite order (Becker & Reed, 2012). Also contributing to selective blockade is the fact that myelinated nerves require blockade of at least three nodes of Ranvier to halt impulse conduction and the increased internodal distance in larger nerves makes these nerves more resistant to blockade (Fink, 1989). Not only fibre‐type but also specific drugs can contribute to differential blockade with bupivacaine (0.125%) reported to have more sensory than motor blockade (Bleyaert, Soetens, Vaes, Steenberge, & Donck, 1979). Ropivacaine and levobupivacaine (Camorcia, Capogna, Berritta, & Columb, 2007) and liposome‐encapsulated bupivacaine (Joshi, Patou, & Kharitonov, 2015) cause less motor blockade than regular bupivacaine. Clinically, higher dosages and/or concentrations of any of these drugs are more likely to cause motor blockade and motor blockade should always be anticipated. This can be concerning if patients cannot use limbs to ambulate following surgery but the motor effects are generally minimal or absent by the time the patient has recovered from anaesthesia to the point that it is ambulatory. Motor blockade and subsequent muscle relaxation may actually be useful in a number of instances, as in fracture reduction.

The speed of onset of action of local anaesthetics is determined by the effect of pKa on the number of lipid soluble molecules at the cell membrane. It is the unionized lipid soluble molecules that can more easily, thus more rapidly, cross into the cell and the pKa of the drug dictates the proportion of molecules that are in an unionized lipid‐soluble state (Berde & Strichartz, 2000; Catterall & Mackie, 2001; Scholz et al., 2004). Drugs like lidocaine with a pKa (7.9) near physiologic pH (7.4) have a fast onset of action, whereas drugs like bupivacaine and ropivacaine (pKa 8.1) have a slower onset of action. An acidic local tissue environment, as might occur with infection, will cause an increased number of local anaesthetic (weak bases) molecules to remain in the unionized state and the onset of action may be slower (Berde & Strichartz, 2000; Catterall & Mackie, 2001; Scholz et al., 2004). Although it is the lipid soluble molecules that cross into the cell, increased lipid solubility of the drug may actually cause slower onset of action since the injected drug will have more uptake into lipid tissues like fat (Gissen, Covino, & Gregus, 1982). The drug is then slowly released from the lipid compartment instead of acting relatively immediately on the nerve, which slows onset, but prolongs duration, of action (Campoy & Read, 2013). While lipid solubility and protein binding are separate attributes, they are also related. Drugs that are more lipid soluble have greater protein binding, which also increases the duration of the block. Thus, drugs with high lipid solubility like bupivacaine have a longer duration of action than those with low lipid solubility like lidocaine. Finally, potency, described as the number of molecules needed to produce a pharmacologic effect (i.e. dose), is also based on lipid solubility, with increased solubility equating to increased potency. These properties (pKa, lipid solubility, protein binding) are inherent to each drug and determined by the chemical structure of that drug (Berde & Strichartz, 2000; Catterall & Mackie, 2001; Scholz et al., 2004).

For all local anaesthetic drugs, the clinical time‐to‐onset, duration‐of‐action and recommended dose may vary slightly between clinical references and the (generally) small variances are often based on practitioner experience. Dose, concentration of drug and volume of injectate can also impact these parameters. The drug information in this manuscript is compiled from several veterinary‐specific references (Campoy & Read, 2013; Duke‐Novakovski, 2016; Lemke, 2007; Rioja Garcia, 2015) and from the clinical experience of the authors. Where available, specific dosing references are provided. The doses listed are total cumulative doses and should be divided between blocks if more than one block is planned. If lidocaine infusions are included in the analgesic protocol, the low end of the local block dose and the low end of the infusion dose should be used to avoid overdosage. However, it is not necessary to include the very small amount of lidocaine typically used on the arytenoids during intubation of cats as part of the total cumulative dose. In adult cats, 2% lidocaine dosed at 0.1 ml (total dose) administered topically on the larynx PLUS 0.1 ml/kg administered intratesticularly produced serum concentrations well below toxic levels (Soltaninejad & Vesal, 2018). In contrast, as mentioned by the reviewer of this manuscript, the dose of a specific product (not used by the authors) is up to 5 mg/kg for arytenoid desensitization. This is a significant contribution and should be considered as part of the total dose.

### 1.2 Adverse effects caused by local anaesthetic drugs

The most serious adverse effects generally occur secondary to rapid IV bolus of a supra‐clinical dose of local anaesthetic drugs (Epstein et al., 2015; Mathews et al., 2014; Rioja Garcia, 2015). Although the drugs (other than lidocaine) are unlikely to be administered rapidly IV, accidental intravascular injection, as would be most likely with poor injection technique, could occur with any block. **Consequently, aspiration should always be used to determine correct needle placement prior to local anaesthetic drug injections.** The incidence of systemic toxicity in veterinary species is not documented, but in the opinions of both the authors and other pain management experts (Epstein et al., 2015; Mathews et al., 2014), it is very low. The incidence of systemic toxicity in humans is 1–7.5 in 10,000 (Auroy et al., 2002; Faccenda & Finucane, 2001). Among local anaesthetics, only lidocaine can be safely administered intravenously. Although commonly used by this route, the administration is not without potential adverse effects. At low serum lidocaine concentrations, inhibitory neuron depression can cause muscle fasciculations, weakness, visual disturbances and can potentially cause cerebral excitation and seizures. At higher concentrations (i.e. overdose), profound central nervous system (CNS) depression with subsequent coma, respiratory arrest and death can occur. Other than bupivacaine, the toxic effects follow a gradation like that just described for lidocaine, with lower dosages causing signs such as mild muscle twitching, and progressing as dosages increase through seizures, unconsciousness, coma, respiratory arrest and cardiovascular collapse (Rioja Garcia, 2015). Bupivacaine is more cardiotoxic and cardiac signs can occur simultaneous with central nervous system signs. Cardiovascular system adverse effects are most commonly associated with a bupivacaine overdose and are due to the higher lipophilicity of bupivacaine and longer duration of sodium channel blockade when compared with other local anaesthetic drugs (Greensmith & Bosseau, 2006). IV boluses of bupivacaine can induce hypotension or cardiovascular collapse, which can be fatal, secondary to blockade of the myocardial conduction system. This is unlikely with liposome‐encapsulated bupivacaine (see more information under specific drugs). Inadvertent IV injections of lipophilic drugs, which includes all local anaesthetics with bupivacaine being the most lipophilic, can be managed by the “lipid rescue” protocol to sequester the lipid soluble drug until it can be cleared (Weinberg, Ripper, Feinstein, & Hoffman, 2003). In lipid rescue a 20% lipid emulsion is infused intravenously as emergency treatment. Although the mechanism of action remains unknown, the presumption is that the injected lipids form a ‘sink’ for the local anaesthetic to bind to, thus decreasing binding to lipid cellular membranes, which decreases the toxic effects (Rothschild, Bern, Oswald, & Weinberg, 2010). Other adverse effects of local anaesthetics include anaphylaxis, which is very rare and primarily associated with esters (e.g. procaine) and drugs containing methylparaben as a preservative. Lidocaine, ropivacaine, mepivacaine and bupivacaine, including liposome‐encapsulated bupivacaine, are amides. Methemoglobinaemia is rare and primarily associated with benzocaine (ester) in cats. The toxic effects are covered in more detail in other references (Rioja Garcia, 2015). Dosages of drugs shown to cause systemic toxicity are listed in the section on individual drugs. If not listed, published data are unavailable.

In addition to systemic adverse effects, nerve damage and specific injection site‐related adverse effects could occur and are discussed in Part 2 of the manuscript (Grubb & Lobprise, 2020). Infections from local anaesthetic injections are an extremely unlikely adverse event because local anaesthetics have a mild antimicrobial effect (Johnson, Saint John, & Dine, 2008). However, micro‐organisms can spread along the needle tract when infected tissues are infiltrated.

### 1.3 Specific local anaesthetic drugs

#### 1.3.1 Lidocaine Hydrochloride (HCl)

BOX 1 **Properties of lidocaine hydrochloride**

▪ Onset‐of‐action: rapid, approximately 1–2 (<5) min
▪ Duration‐of‐action: 60–120 min
▪ **Recommended dose:** 4–6 mg/kg (dog); 2–4 mg/kg (cat)
▪ Toxic dose: The cumulative IV dose for CNS toxicity resulting in convulsive activity in conscious dogs was 20 mg/kg (Feldman, Arthur, & Covino, 1989) or 22 mg/kg (Liu, Feldman, Giasi, Patterson, & Covino, 1983) and the IV dose resulting in death from cardiovascular toxicity in pentobarbital anaesthetized dogs was 80 mg/kg (Liu, Feldman, Covino, Giasi, & Covino, 1982) or 127 mg/kg in fentanyl/midazolam anaesthetized dogs (Groban, Deal, Vernon, James, & Butterworth, 2001). In cats, the mean convulsant dose was 11.7 ± 4.6 IV and 47.3 ± 8.6 IV for cardiovascular collapse (Chadwick, 1985).

#### 1.3.2 Bupivacaine Hydrochloride (HCl)

BOX 2 **Properties of bupivacaine hydrochloride**

▪ Onset‐of‐action: approximately 2–5 min for first onset and 5–10 min for full blockade (potentially but uncommonly up to 20 min in large nerves).
▪ Duration‐of‐action: 4–6 hr for diffusion techniques, potentially 6–8 hr when injected into a dental foramen (Lantz, 2003). A range of 4–12 hr has been reported (Campoy & Read, 2013).
  • The duration may be longer than previously reported, exceeding 24 hr in one dental study (Snyder & Snyder, 2016).
▪ **Recommended dose:** 1–2 mg/kg (dog); 1 mg/kg (cat)
▪ Toxic dose: The cumulative IV dose for CNS toxicity resulting in convulsive activity in conscious dogs was 4.3 mg/kg (Feldman et al., 1989) or 8 mg/kg (Liu et al., 1983) and the IV dose resulting in death from cardiovascular toxicity in pentobarbital anaesthetized dogs was 20 mg/kg (Liu et al., 1982) or 22 mg/kg in fentanyl/midazolam anaesthetized dogs (Groban et al., 2001). In cats, the mean convulsant dose was 3.8 ± 1.0 mg/kg IV and 18.4+/4.9 IV for cardiovascular collapse (Chadwick, 1985).
▪ Levobupivacaine has properties and dosages very similar to that of bupivacaine but is less cardiotoxic than bupivacaine in dogs, requiring 27 mg/kg to produce cardiovascular collapse in fentanyl/midazolam anaesthetized dogs (Groban et al., 2001), and may be less likely than bupivacaine to cause motor blockade in dogs (Gomez Segura, Menafro, García‐Fernández, Murillo, & Parodi, 2009). Levobupivacaine has been reported for clinical use in cats (Vettorato & Corletto, 2016). The drug is not currently used by authors of this manuscript.

#### 1.3.3 Ropivacaine hydrochloride (HCl)

BOX 3 **Properties of ropivacaine hydrochloride**

▪ Onset‐of‐action: approximately 5–10 min
▪ Duration‐of‐action: 4–6 hr for diffusion techniques. A range of 5–8 hr has been reported (Campoy & Read, 2013).
▪ **Recommended dose:** 1–3 mg/kg (dog); 1–2 mg/kg (cat)
▪ Toxic dose: The cumulative IV dose for CNS toxicity resulting in convulsive activity in conscious dogs was 4.88 mg/kg (Feldman et al., 1989). Cardiovascular collapse was caused by 42 mg/kg IV in fentanyl/midazolam anaesthetized dogs (Groban et al., 2001). The latter data illustrate the increased safety and decreased cardiovascular adverse effects when compared to lidocaine or bupivacaine.
▪ Ropivacaine is structurally similar to bupivacaine but is less cardiotoxic and less likely to cause motor dysfunction (Camorcia et al., 2007).

#### 1.3.4 Mepivacaine hydrochloride (HCl)

BOX 4 **Properties of mepivacaine hydrochloride**

▪ Onset‐of‐action: 2–5 min
▪ Duration‐of‐action: 2–3 hr for soft tissue and 0.5–1 hr for pulp desensitization in dental procedures (Lantz, 2003).
▪ **Recommended dose:** 5–6 mg/kg (dog); 2–3 mg/kg (cat)
▪ Toxic dose: The IV dose resulting in death from cardiovascular toxicity in pentobarbital anaesthetized dogs was 80 mg/kg (Liu et al., 1982).

#### 1.3.5 Articaine hydrochloride (HCl)

Articaine is widely used in human dentistry and is characterized by a quicker onset and shorter elimination compared to other local anaesthetic drugs (Lasemi et al., 2015) so re‐injection is likely safer, if needed (Johansen, 2004; Vree & Gielen, 2005). It has better diffusion through soft tissue and bone than other local anaesthetics, resulting in postextraction drug concentrations that are higher in tooth alveolus than in systemic circulation (Vree & Gielen, 2005). The drug is used anecdotally in veterinary medicine and by the author but there are no studies on its use in dogs and cats.

#### 1.3.6 Bupivacaine liposome‐encapsulated injectable suspension

Bupivacaine supplied as a liposome‐encapsulated injectable suspension (abbreviated as BLIS in this manuscript; NOCITA®), is the newest of the local anaesthetics and is approved for veterinary use, although currently only in the US. In 2016, the US Food and Drug Administration (FDA) approved single‐dose infiltration of BLIS (13.3 mg/mL) into the surgical site to provide local postoperative analgesia for cranial crucial ligament surgery in dogs (NOCITA® product insert website). In 2018, BLIS was approved for use as a peripheral nerve block for postoperative regional analgesia following onychectomy in cats (NOCITA® product insert website). NOTE: Onychectomy is not supported by the authors nor by the company producing NOCITA®. However, this surgery is accepted by the FDA as causing recognizable pain in cats, thus it is often used to test analgesic modalities submitted for regulatory approval. A review of potential BLIS uses as described in the veterinary and human literature has been published (Lascelles & Kirkby‐Shaw, 2016).

BOX 5 **Properties of bupivacaine liposome‐encapsulated suspension**

▪ Onset‐of‐action: Within 2–5 min (in humans [Apseloff, Onel, & Patou, 2013]; not reported in animals)
▪ Duration‐of‐action: Up to 72 hr (Lascelles et al., 2016; NOCITA® product insert website),
▪ **Label‐approved dose:** 5.3 mg/kg (0.4 ml/kg) total dose in the dog; 5.3 mg/kg per forelimb (0.4 ml/kg per forelimb) for a total dose of 10.6 mg/kg in the cat (NOCITA® product insert website).
  - If a larger volume of drug is required to infiltrate the tissue area, according to the prescribing information for dogs, BLIS may be volume expanded 1:1 with sterile 0.9% normal saline or Lactated Ringer's solution to ensure adequate coverage of the infiltration area without reduced efficacy. Water or other hypotonic solutions may disrupt the liposomes and should not be used. BLIS should not be mixed with lidocaine as this can cause significant disruption of the liposomes (Kharitonov, 2014). However, an admixture of BLIS with bupivacaine HCl is proposed to decrease the onset time of BLIS in humans (Eppstein & Sakamoto, 2016) and the guideline from human medicine is to mix no more than an equal volume (1:1) of 0.5% bupivacaine HCl:liposomal bupivacaine. Mixing has not been studied in animals but the fast onset of BLIS, as reported in humans (Apseloff et al., 2013), may preclude the need for this technique.
▪ Toxic dose: Unknown, but the maximum doses at which no meaningful adverse events were observed after IV bolus in conscious dogs were higher with BLIS (4.5 mg/kg) than bupivacaine HCl (0.75 mg/kg; Joshi et al., 2015). In the same study, the free‐fraction of serum bupivacaine was similar between both drugs, even though the dosages were drastically different. It is likely that the slow bupivacaine release due to liposomal encapsulation results in lower systemic exposure and decreased incidence of adverse effects (Joshi et al., 2015).
  - BLIS (up to 40 mg total dose per dog) caused less motor blockade than bupivacaine HCl (15 mg total dose per dog) and caused no spinal cord damage when administered epidurally or intrathecally in dogs (Joshi et al., 2015). However, the efficacy of the dose administered in that study was not evaluated.

The injection technique for BLIS is slightly different than that used for other local anaesthetic drugs. When used for tissue infiltration, it is methodically injected into the surgery or wound site tissue during wound/incision closure (currently used for a multitude of wounds/incisions but is off‐label except for closure of incision for stifle surgery) using a 25‐gauge needle or larger since smaller‐bore needles can disrupt the liposomes (NOCITA® product insert website)**.** Injection at closure prevents liposome disruption during surgical tissue manipulation. For pre‐emptive blockade in humans, regular bupivacaine or lidocaine has been used at the incision site with BLIS administered at closure (Kharitonov, 2014). The authors have used this technique but there are no published data regarding this in animals. When used for nerve blocks that are not at the incision site (currently used for a multitude of nerve blocks but is off‐label except for blockade of the radius/ulnar/musculocutaneous nerves), pre‐emptive administration is possible since the liposomes at the remote location will not be disrupted during the surgical incision (see discussion of specific blocks in Part 2 for more information) (Grubb & Lobprise, 2020). Due to their relatively large size, the liposomes release bupivacaine locally rather than diffusing throughout the tissue, thus, BLIS is not likely to be highly effective for ‘splash’ (i.e. ‘squirting’ local anaesthetics into the incision or wound) blocks. A video of the injection technique and mechanism of action of the slow‐release from the liposomes is available on the product website (NOCITA® injection technique website).

Logistics of use: BLIS vials should be punctured once and individual doses drawn into sterile syringes, which can be kept at room temperature for up to 4 hr, according to the label. The drug contains no preservative so sterility cannot be guaranteed for > 4 hr and the liposome's duration‐of‐stability after exposure to air for > 4 hr in the broached vial is unknown. Rapid liposomal break down and release of bupivacaine does not result in a toxic concentration of bupivacaine, but will likely decrease the 72‐hr duration. However, once injected into tissue, the liposomes are stable and gradually break down over 72 hr, enacting the extended release of bupivacaine. More detail on handling BLIS is available in the prescribing information at the product website (NOCITA® product insert website).

The up‐to‐72‐hr duration of BLIS‐induced analgesia is an important advantage for postoperative pain control. As stated, inadequately treated postoperative pain is the leading cause of chronic pain development in humans (de Brito et al., 2012; Puolakka et al., 2010; Voscopoulos & Lema, 2010) and, presumably, in animals because of the similarity of the pain pathway. Unfortunately, there are few drug choices for multimodal moderate duration postoperative pain treatment, especially after the patient is discharged from the hospital, eliminating choices such as IV infusions. Anti‐inflammatory drugs are used to control pain in this time period but may not provide adequate analgesia when used alone for treatment of moderate‐to‐severe pain. Opioids used perioperatively can also decrease chronic pain development but dispensing controlled drugs for at‐home use can be complicated and opioid‐induced adverse effects, such as sedation, nausea and vomiting, are often concerning to the owners. Long‐duration local and regional anaesthetic blockade could potentially fill the analgesic gap during this time period.

### 1.4 Adjuvants for perineural injection with local anaesthetic drugs

#### 1.4.1 Opioids

Buprenorphine has been used to prolong the duration of local blockade in humans (Modi, Rastogi, & Kumar, 2009) and buprenorphine (0.004 mg/kg) added to bupivacaine in the epidural space provided up to 24‐hr of pain relief in two‐thirds of dogs undergoing stifle arthroplasty (Bartel et al., 2016). A combination of 0.1 ml buprenorphine and 0.3 ml 0.5% bupivacaine for infraorbital nerve blocks provided analgesia for > 24 hr in some dogs (Snyder & Snyder, 2016). The authors use 0.003–0.004 mg/kg combined with a local anaesthetic for perineural injection. Other opioid adjuvants appear to be less successful at extending analgesic duration of the local anaesthetic.

#### 1.4.2 Alpha‐2 agonists

The addition of 0.01 mg/kg medetomidine (Lamont & Lemke, 2008) added to local anaesthetic drugs has been shown to increase the duration of perineural local anaesthetic blockade in dogs. Dexmedetomidine has been shown to prolong the duration of local anaesthetic blockade in humans (Wu et al., 2014) and provided up to 24 hr of analgesia when dosed at 0.0001 mg/kg added to 0.5 mg/kg bupivacaine for femoral nerve blocks in dogs (Bartel et al., 2016). The authors use 0.0001 mg/kg combined with a local anaesthetic for perineural injection.

## 2 CONCLUSION

By acting directly on the propagation of nerve impulses, local anaesthetic drugs provide a unique mechanism for analgesia. Blockade of nociceptive/pain impulses provides profound analgesia intra‐ and postoperatively with minimal risk of adverse effects often associated with some systemically administered analgesic drugs. With local blockade, inhalant dosages intraoperatively and opioid dosages both intra‐ and postoperatively can generally be reduced, which promotes a faster recovery from anaesthesia and discharge of a pet that is more alert and interactive with its owner. The local anaesthetic drugs used most commonly in veterinary medicine include lidocaine, bupivacaine, ropivacaine and, more recently, liposome‐encapsulated bupivacaine.

## CONFLICT OF INTEREST

Although manuscript preparation was supported by Aratana Therapeutics, who manufactures liposome‐encapsulated bupivacaine (NOCITA®), the authors feel that the information in the manuscript is a balanced view of the use of all local anaesthetics with detailed information on liposome‐encapsulated bupivacaine since it is a new product. The authors have no other conflicts.

## Ethical statement

The authors confirm that the ethical policies of the journal, as noted on the journal's author guidelines page, have been adhered to. No ethical approval was required as this is a review article with no original research data.

## References

[vms3219-bib-0001] Aguiar, J. , Chebroux, A. , Martinez‐Taboada, F. , & Leece, E. A. (2015). Analgesic effects of maxillary and inferior alveolar nerve blocks in cats undergoing dental extractions. Journal of Feline Medicine & Surgery, 17(2), 110–116. 10.1177/1098612X14533551 24820999 PMC10816425

[vms3219-bib-0002] Althaus, A. , Arránz, B. O. , Moser, K. H. , Lux, E. A. , Weber, F. , Neugebauer, E. , & Simanski, C. (2018). Postoperative Pain Trajectories and Pain Chronification‐an Empirical Typology of Pain Patients. Pain Med, 19(12), 2536–2545. 10.1093/pm/pny099 29800281

[vms3219-bib-0003] Apseloff, G. , Onel, E. , & Patou, G. (2013). Time to onset of analgesia following local infiltration of liposome bupivacaine in healthy volunteers: A randomized, single‐blind, sequential cohort, crossover study. International Journal of Clinical Pharmacology and Therapeutics, 51(5), 367–373. 10.5414/CP201775 23458225

[vms3219-bib-0004] Auroy, Y. , Benhamou, D. , Bargues, L. , Ecoffey, C. , Falissard, B. , Mercier, F. J. , … Samii, K. (2002). Major complications of regional anesthesia France: The SOS Regional Anesthesia. Hotline Service. 10.1097/00000542-200211000-00034 12411815

[vms3219-bib-0005] Bartel, A. K. , Campoy, L. , Martin‐Flores, M. , Gleed, R. D. , Walker, K. J. , Scanapico, C. E. , & Reichard, A. B. (2016). Comparison of bupivacaine and dexmedetomidine femoral and sciatic nerve blocks with bupivacaine and buprenorphine epidural injection for stifle arthroplasty in dogs. Veterinary Anaesthesia and Analgesia, 43(4), 435–443. 10.1111/vaa.12318 26529670

[vms3219-bib-0006] Becker, D. E. , & Reed, K. L. (2012). Local Anesthetics: Review of. Pharmacological Considerations. Anesthesia Progress, 59(2), 90–102. 10.2344/0003-3006-59.2.90 22822998 PMC3403589

[vms3219-bib-0007] Benito, J. , Monterrey, B. , Lavoie, A. M. , Beauchamp, G. , Lascelles, B. D. X. , & Steagall, P. V. (2016). Analgesic efficacy of intraperitoneal administration of bupivacaine in cats. Journal of Feline Medicine & Surgery, 18(11), 906–912. 10.1177/1098612X15610162 26467541 PMC11132218

[vms3219-bib-0008] Berde, C. B. , & Strichartz, G. R. (2000). Local anesthetics Miller R. D., Cucchiara R. F., Miller E. D., Reves J. G., Roizen M. F., & Savarese J. J. (Eds), Anesthesia, 5th ed. (pp. 491–522). Philadelphia, PA: Churchill Livingstone (Elsevier).

[vms3219-bib-0009] Bergese, S. D. , Ramamoorthy, S. , Paton, G. , Bramlett, K. , Gorfine, S. R. , & Candiotti, K. A. (2012). Efficacy profile of liposome bupivacaine, a novel formulation of bupivacaine for postsurgical analgesia. Journal of Pain Research, 5, 107–116. 10.2147/JPR.S30861 22570563 PMC3346068

[vms3219-bib-0010] Blanco, R. , Ansari, T. , & Girgis, E. (2015). Quadratus lumborum block for postoperative pain after caesarean section: A randomised controlled trial. European Journal of Anaesthesiology, 32(11), 812–818. 10.1097/EJA.0000000000000299 26225500

[vms3219-bib-0011] Bleyaert, A. , Soetens, M. , Vaes, L. , Van Steenberge, A. L. , & Van der Donck, A. (1979). Bupivacaine, 0.125 per cent, in obstetric epidural analgesia: Experience in three thousand cases. Anesthesiology, 51(5), 435–438. 496059 10.1097/00000542-197911000-00013

[vms3219-bib-0012] Boerboom, S. L. , de Haes, A. , Vd, W. L. , Aarts, E. O. , Janssen, I. M. C. , Geurts, J. W. , & Kamphuis, E. T. (2018). Preperitoneal bupivacaine infiltration reduces postoperative opioid consumption, acute pain, and chronic postsurgical pain after bariatric surgery: A randomized controlled trial. Obesity Surgery, 28(10), 3102–3110. 10.1007/s11695-018-3341-6 29926357

[vms3219-bib-0013] Camorcia, M. , Capogna, G. , Berritta, C. , & Columb, M. O. (2007). The relative potencies for motor block after intrathecal ropivacaine, levobupivacaine, and bupivacaine. Anesthesia and Analgesia, 104(4), 904–907. 10.1213/01.ane.0000256912.54023.79 17377104

[vms3219-bib-0014] Campoy, L. , & Read, M. R. (Eds.) (2013). Small animal regional anesthesia and analgesia. Ames, IA: Wiley‐Blackwell.

[vms3219-bib-0015] Candiotti, K. (2012). Liposomal bupivacaine: An innovative nonopioid local analgesic for the management of postsurgical pain. Pharmacotherapy, 32(9 Suppl), 195–265. 10.1002/j.1875-9114.2012.01183.x 22956491

[vms3219-bib-0016] Carpenter, R. E. , Wilson, D. V. , & Evans, A. T. (2004). Evaluation of intraperitoneal and incisional lidocaine or bupivacaine for analgesia following ovariohysterectomy in the dog. Veterinary Anaesthesia & Analgesia, 31(1), 46–52. 10.1111/j.1467-2995.2004.00137.x 14756753

[vms3219-bib-0017] Catterall, W. A. , & Mackie, K. (2001). Local Anesthetics In: Hardman J. G., Limbird L. E., & Gilman A. G. (Eds), Goodman & Gilman’s The Pharmacologic Basis of Therapeutics, 10th ed. (pp. 367–384). New York, NY: McGraw‐Hill.

[vms3219-bib-0018] Chadwick, H. S. (1985). Toxicity and resuscitation in lidocaine‐ or bupivacaine‐infused cats. Anesthesiology, 63(4), 385–390. 10.1097/00000542-198510000-00007 4037401

[vms3219-bib-0019] Conzemius, M. G. , Brockman, D. J. , King, L. G. , & Perkowski, S. Z. (1994). Analgesia in dogs after intercostal thoracotomy: A clinical trial comparing intravenous buprenorphine and interpleural bupivacaine. Veterinary Surgery, 23(4), 291–298. 10.1111/j.1532-950X.1994.tb00487.x 8091633

[vms3219-bib-0020] de Brito Cancado, T. O. , Omanis, M. , Ashmawi, H. A. , & Torres, M. I. (2012). Chronic pain after cesarean section. Influence of anesthetic/surgical technique and postoperative analgesia. Brazilian Journal of Anesthesiology, 62(6), 762–774. 23176985 10.1016/S0034-7094(12)70177-0

[vms3219-bib-0021] Duke‐Novakovski, T. (2016). Pain management II: Local and regional anaesthetic techniques In Duke‐Novakovski T., deVries M., & Seymore C. (Eds.), Manual of Canine and Feline Anaesthesia and Analgesia, 3rd ed. (pp. 143–158). Gloucester, UK: BSAVA.

[vms3219-bib-0022] Eppstein, A. C. , & Sakamoto, B. (2016). The novel use of different bupivacaine preparations with combined regional techniques for postoperative pain management in non‐opioid‐based laparoscopic inguinal herniorrhaphy. Journal of Clinical Anesthesia, 34, 403–406. 10.1016/j.jclinane.2016.05.011 27687421

[vms3219-bib-0023] Epstein, M. , Rodan, I. , Griffenhagen, G. , Kadrlik, J. , Petty, M. , Robertson, S. , & Simpson, W. (2015). AAHA/AAFP Pain Management Guidelines for Dogs and Cats. Journal of the American Animal Hospital Association, 51(2), 67–84. 10.5326/JAAHA-MS-7331 25764070

[vms3219-bib-0024] Faccenda, K. A. , & Finucane, B. T. (2001). Complications of regional anaesthesia Incidence and prevention. Drug Safety, 24(6), 413–442. 10.2165/00002018-200124060-00002 11368250

[vms3219-bib-0025] Feldman, H. S. , Arthur, G. R. , & Covino, B. G. (1989). Comparative systemic toxicity of convulsant and supraconvulsant doses of intravenous ropivacaine, bupivacaine and lidocaine in the conscious dog. Anesthesia and Analgesia, 69, 794–801. 10.1213/00000539-198912000-00019 2511782

[vms3219-bib-0026] Fink, B. R. . (1989) Mechanisms of differential axial blockade in epidural and subarachnoid anesthesia. Anesthesiology 70(5), 851‐858. 2719320 10.1097/00000542-198905000-00023

[vms3219-bib-0027] Flecknell, P. A. , Kirk, A. J. , Liles, J. H. , Hayes, P. H. , & Dark, J. H. (1991). Post‐operative analgesia following thoracotomy in the dog: An evaluation of the effects of bupivacaine intercostal nerve block and nalbuphine on respiratory function. Laboratory Animals, 25(4), 319–324. 10.1258/002367791780810029 1753691

[vms3219-bib-0028] Gasser, H. S. , & Erlanger, J. (1929). Role of fiber size in establishment of nerve block by pressure and cocaine. American Journal of Physiology, 88, 581–581.

[vms3219-bib-0029] Gissen, A. J. , Covino, B. G. , & Gregus, J. (1982). Differential sensitivity of fast and slow fibers n mammalian nerve. III. Effect of etidocaine and bupivacaine on fast/slow fibers. Anesthesia and Analgesia, 61, 570–575. 6283948

[vms3219-bib-0030] Gomez de Segura, I. A. , Menafro, A. , García‐Fernández, P. , Murillo, S. , & Parodi, E. M. (2009). Analgesic and motor‐blocking action of epidurally administered levobupivacaine or bupivacaine in the conscious dog. Veterinary Anaesthesia and Analgesia, 36(5), 485–494. 10.1111/j.1467-2995.2009.00469.x 19508452

[vms3219-bib-0031] Greensmith, J. E. , & Bosseau, M. W. (2006). Complications of regional anesthesia. Current Opinion in Anaesthesiology, 19, 531–537. 10.1097/01.aco.0000245280.99786.a3 16960487

[vms3219-bib-0032] Groban, L. , Deal, D. D. , Vernon, J. C. , James, R. L. , & Butterworth, J. (2001). Cardiac resuscitation after incremental overdosage with lidocaine, bupivacaine, levobupivacaine, and ropivacaine in anesthetized dogs. Anesthesia and Analgesia, 92(2), 37–43. 10.1097/00000539-200101000-00008 11133597

[vms3219-bib-0033] Grubb, T. , Lobprise, H. (2020). Local and regional anaesthesia in dogs and cats: Descriptions of specific local and regional techniques (Part 2). Veterinary Medicine and Science, in press, 10.1002/vms3.218 PMC7196680 31965749

[vms3219-bib-0034] Jin, J. , Peng, L. , Chen, Q. , Zhang, D. , Ren, L. , Qin, P. , & Min, S. (2016). Prevalence and risk factors for chronic pain following cesarean section: A prospective study. BMC Anesthesiol, 16(1), 99 10.1186/s12871-016-0270-6 27756207 PMC5069795

[vms3219-bib-0035] Johansen, O. (2004). Comparison of articaine and lidocaine used as dental local anesthetics. Project Thesis. Oslo: Institute of Clinical Dentistry.

[vms3219-bib-0036] Johnson, S. M. , Saint John, B. E. , & Dine, A. (2008). Local Anesthetics as Antimicrobial Agents: A Review. Surgical Infections, 9(2), 2005–2213. 10.1089/sur.2007.036 18426354

[vms3219-bib-0037] Joshi, G. P. , Patou, G. , & Kharitonov, V. (2015). The safety of liposome bupivacaine following various routes of administration in animals. Journal of Pain Research, 8, 781–789. 10.2147/JPR.S85424 26586964 PMC4634838

[vms3219-bib-0038] Kharitonov, V. (2014). A review of the compatibility of liposome bupivacaine with other drug products and commonly used implant materials. Postgraduate Medicine, 126(1), 129–138. 10.3810/pgm.2014.01.2733 24393760

[vms3219-bib-0039] Kim, R. (2017). Anesthetic technique and cancer recurrence in oncologic surgery: unraveling the puzzle. Cancer and Metastasis Reviews 36(1), 159‐177. 27866303 10.1007/s10555-016-9647-8

[vms3219-bib-0040] Kona‐Boun, J. J. , Cuvelliez, S. , & Troncy, E. (2006). Evaluation of epidural administration of morphine or morphine and bupivacaine for postoperative analgesia after premedication with an opioid analgesic and orthopedic surgery in dogs. Journal of the American Veterinary Medical Association, 229(7), 1103–1112. 10.2460/javma.229.7.1103 17014357

[vms3219-bib-0041] Lamont, L. A. , & Lemke, K. A. (2008). The effects of medetomidine on radial nerve blockade with mepivacaine in dogs. Veterinary Anaesthesia and Analgesia, 35(1), 62–68. 10.1111/j.1467-2995.2007.00349.x 17565571

[vms3219-bib-0042] Lantz, G. C. (2003). Regional anesthesia for dentistry and oral surgery. Journal of Veterinary Dentistry, 20(3), 181–186. 10.1177/089875640302000306 14705435

[vms3219-bib-0043] Lascelles, B. D. X. , & Kirkby, S. K. (2016). An extended release local anaesthetic: Potential for future use in veterinary surgical patients? Veterinary Medicine & Science, 2(4), 229–238. 10.1002/vms3.43 29067198 PMC5645851

[vms3219-bib-0044] Lascelles, B. D. X. , Rausch‐Derra, L. C. , Wofford, J. A. , & Huebner, M. (2016). Pilot, randomized, placebo‐controlled clinical field study to evaluate the effectiveness of bupivacaine liposome injectable suspension for the provision of post‐surgical analgesia in dogs undergoing stifle surgery. BMC Veterinary Research, 12(1), 168. 27531019 10.1186/s12917-016-0798-1 PMC4988028

[vms3219-bib-0045] Lasemi, E. , Sezavar, M. , Habibi, L. , Hemmat, S. , Sarkarat, F. , & Nematollahi, Z. (2015). Articaine (4%) with epinephrine (1:100,000 or 1:200,000) in inferior alveolar nerve block: Effects on the vital signs and onset, and duration of anesthesia. Journal of Dental Anesthesia and Pain Medicine, 15(4), 201–205. 10.17245/jdapm.2015.15.4.201 28879280 PMC5564155

[vms3219-bib-0046] Lemke, K. (2007). Pain management II: Local and regional anaesthetic techniques. Manual of Canine and Feline Anaesthesia and Analgesia, 2nd. ed. (pp. 104–114). Gloucester, UK: BSAVA.

[vms3219-bib-0047] Liu, P. , Feldman, H. S. , Covino, B. M. , Giasi, R. , & Covino, B. G. (1982). Acute cardiovascular toxicity of intravenous amide local anesthetics in anesthetized ventilated dogs. Anesthesia and Analgesia, 61(4), 317–322. 7199848

[vms3219-bib-0048] Liu, P. L. , Feldman, H. S. , Giasi, R. , Patterson, M. K. , & Covino, B. G. (1983). Comparative CNS toxicity of lidocaine, etidocaine, bupivacaine, and tetracaine in awake dogs following rapid intravenous administration. Anesthesia and Analgesia, 62, 375–379. 10.1213/00000539-198304000-00001 6829942

[vms3219-bib-0049] Lombardi, A. V. Jr (2014). Recent advances in incorporation of local analgesics in postsurgical pain pathways. American Journal of Orthopedics, 43(10 Suppl), S2–5. 25303456

[vms3219-bib-0050] Malik, O. , Kaye, A. D. , Belani, K. , & Urman, R. D. (2017). Emergency roles of liposomal bupivacaine in anesthesia practice. Journal of Anaesthesiology and Clinical Pharmacology, 33(2), 151–156. 10.4103/joacp.JOACP_375_15 PMC5520585 28781438

[vms3219-bib-0051] Marques, E. M. , Jones, H. E. , Elvers, K. T. , Pyke, M. , Blom, A. W. , & Beswisk, A. D. (2014). Local anaesthetic infiltration for peri‐operative pain control in total hip and knee replacement: Systematic review and meta‐analyses of short‐ and long‐term effectiveness. BMC Musculoskeletal Disorders, 15, 220 10.1186/1471-2474-15-220 24996539 PMC4118275

[vms3219-bib-0052] Mathews, K. , Kronen, P. W. , Lascelles, D. , Nolan, A. , Robertson, S. , Steagall, P. V. M. , … Yamashita, K. (2014). Guidelines for recognition, assessment and treatment of pain: WSAVA Global Pain Council. Journal of Small Animal Practice, 55(6), E10–68. 10.1111/jsap.12200 24841489

[vms3219-bib-0053] McMillan, M. W. , Seymour, C. J. , & Brearley, J. C. (2012). Effect of intratesticular lidocaine on isoflurane requirements in dogs undergoing routine castration. Journal of Small Animal Practice, 53(7), 393–397. 10.1111/j.1748-5827.2012.01233.x 22747731

[vms3219-bib-0054] Modi, M. , Rastogi, S. , & Kumar, A. (2009). Buprenorphine with bupivacaine for intraoral nerve blocks to provide postoperative analgesia in outpatients after minor oral surgery. Journal of Oral & Maxillofacial Surgery, 67(12), 2571–2576. 10.1016/j.joms.2009.07.014 19925973

[vms3219-bib-0055] Mosing, M. , Reich, H. , & Moens, Y. (2010). Clinical evaluation of the anaesthetic sparing effect of brachial plexus block in cats. Veterinary Anaesthesia & Analgesia, 37(2), 154–161. 10.1111/j.1467-2995.2009.00509.x 20230566

[vms3219-bib-0056] Myrna, K. E. , Bentley, E. , & Smith, L. J. (2010). Effectiveness of injection of local anesthetic into the retrobulbar space for postoperative analgesia following eye enucleation in dogs. Journal of the American Veterinary Medical Association, 237(2), 174–177. 10.2460/javma.237.2.174 20632790

[vms3219-bib-0057] NOCITA® (bupivacaine liposome injectable suspension) (2018a). Injection technique Leawood, KS. Aratana Therapeutics. https://nocita.aratana.com/dogs/dosing-administration/.

[vms3219-bib-0058] NOCITA® (bupivacaine liposome injectable suspension) (2018b). Package Insert. Leawood, KS. Aratana Therapeutics. https://www.aratana.com/wp-content/uploads/2018/08/NOCITA-Package-Insert.pdf.

[vms3219-bib-0059] Perez, T. E. , Grubb, T. L. , Greene, S. A. , Meyer, S. , Valdez, N. , Bingman, J. , & Farnsworth, R. (2013). Effects of intratesticular injection of bupivacaine and epidural administration of morphine in dogs undergoing castration. Journal of the American Veterinary Medical Association, 242(5), 631–642. 10.2460/javma.242.5.631 23402410

[vms3219-bib-0060] Puolakka, P. A. , Roraius, M. G. , Roviola, M. , Puolakka, T. J. , Nordhausen, K. , & Lindgren, L. (2010). Persistent pain following knee arthroplasty. European Journal of Anaesthesiology, 27(5), 455–460. 10.1097/EJA.0b013e328335b31c 20299989

[vms3219-bib-0061] Rashiq, S. , & Dick, B. D. (2014). Post‐surgical pain syndromes: A review for the non‐pain specialist. Canadian Journal of Anaesthesia, 61(2), 123–130. 10.1007/s12630-013-0072-y 24185829

[vms3219-bib-0062] Rioja, G. E. (2015). Local anesthetics; In: Grimm K. A., Lamont L. A., Tranquilli W. J., Greene S. A., & Robertson S. A. (Eds.), Veterinary Anesthesia and Analgesia: The Fifth Edition of Lumb and Jones (332–354). Ames, IA: John Wiley & Sons.

[vms3219-bib-0063] Rodríguez, P. M. , González, F. J. , Sabaté, S. , García, M. , Lamas, C. , Font, A. , … Hoffmann, R. (2018). Low‐concentration distal nerve blocks with 0.125% levobupivacaine versus systemic analgesia for ambulatory trapeziectomy performed under axillary block: A randomized controlled trial. Minerva Anestesiologica, 84(11), 1261–1269. 29405670 10.23736/S0375-9393.18.12291-7

[vms3219-bib-0064] Rothschild, L. , Bern, S. , Oswald, S. , & Weinberg, G. (2010). Intravenous lipid emulsion in clinical toxicology. Scandinavian Journal of Trauma, Resuscitation and Emergency Medicine, 18, 51–59. 10.1186/1757-7241-18-51 PMC2958894 20923546

[vms3219-bib-0065] Savvas, I. , Papazoglou, L. G. , Kazakos, G. , Anagnostou, T. , Tsioli, V. , & Raptopoulos, D. (2008). Incisional block with bupivacaine for analgesia after celiotomy in dogs. Journal of the American Animal Hospital Association, 44(2), 60–66. 10.5326/0440060 18316441

[vms3219-bib-0066] Scholz, A. M. , Salinas, F. V. , & Spencer, L. L. (2004). Analgesics: Ion channel ligands/sodium channel blockers/local anesthetics In: Evers A. S., & Maze M. (Eds.), Anesthetic Pharmacology: Physiologic Principles and Clinical Practice (pp. 507–538). Philadelphia, PA: Churchill Livingstone (Elsevier).

[vms3219-bib-0067] Snyder, C. J. , & Snyder, L. B. (2013). Effect of mepivacaine in an infraorbital nerve block on minimum alveolar concentration of isoflurane in clinically normal anesthetized dogs undergoing a modified form of dental dolorimetry. Journal of the American Veterinary Medical Association, 242(2), 199–204. 10.2460/javma.242.2.199 23276096

[vms3219-bib-0068] Snyder, L. B. , & Snyder, C. J. (2016). Effects of buprenorphine added to bupivacaine infraorbital nerve blocks on isoflurane minimum alveolar concentration using a model for acute dental/oral surgical pain in dogs. Journal of Veterinary Dentistry, 33(2), 90–96. 10.1177/0898756416657232 28326977

[vms3219-bib-0069] Soltaninejad, H. , & Vesal, N. (2018). Plasma concentrations of lidocaine following laryngeal administration or laryngeal and intratesticular administration in cats. American Journal of Veterinary Research, 79(6), 614–620. 10.2460/ajvr.79.6.614 30085855

[vms3219-bib-0070] Stokes, A. L. , Adhikary, S. D. , Quintili, A. , Puleo, F. J. , Choi, C. S. , Hollenbeak, C. S. , & Messaris, E. (2017). Liposomal bupivacaine use in transversus abdominis plane blocks reduces pain and postoperative intravenous opioid requirement after colorectal surgery. Diseases of the Colon and Rectum, 60(2), 170–177. 10.1097/DCR.0000000000000747 28059913

[vms3219-bib-0071] Vettorato, E. , & Corletto, F. (2016). Retrospective assessment of peripheral nerve block techniques used in cats undergoing hindlimb orthopaedic surgery. Journal of Feline Medicine and Surgery, 18(10), 826–833. 10.1177/1098612X15598185 26239941 PMC11112211

[vms3219-bib-0072] Voscopoulos, C. , & Lema, M. (2010). When does acute pain become chronic? British Journal of Anaesthesia, 105(Suppl 1), i69–85. 10.1093/bja/aeq323 21148657

[vms3219-bib-0073] Vree, T. B. , & Gielen, M. J. M. (2005). Clinical pharmacology and the use of articaine for local and regional anaesthesia. Best Practice & Research Clinical Anaesthesiology, 19(2), 293–308. 10.1016/j.bpa.2004.12.006 15966499

[vms3219-bib-0074] Weinberg, G. , Ripper, R. , Feinstein, D. L. , & Hoffman, W. (2003). Lipid emulsion infusion rescues dogs from bupivacaine‐induced cardiac toxicity. Regional Anaesthesia & Pain Medicine, 28(3), 198–202. 10.1097/00115550-200305000-00005 12772136

[vms3219-bib-0075] Wenger, S. , Moens, Y. , Jäggin, N. , & Schatzmann, U. (2005). Evaluation of the analgesic effect of lidocaine and bupivacaine used to provide a brachial plexus block for forelimb surgery in 10 dogs. The Veterinary Record, 156(20), 639–642. 10.1136/vr.156.20.639 15894729

[vms3219-bib-0076] Wu, H. H. , Wang, H. T. , Jin, J. J. , Cui, G. B. , Zhou, K. C. , Chen, Y. , … Wang, W. (2014). Does dexmedetomidine as a neuraxial adjuvant facilitate better anesthesia and analgesia? A systematic review and meta‐analysis. PLoS ONE, 9(3), e93114. 24671181 10.1371/journal.pone.0093114 PMC3966844

[vms3219-bib-0077] Yilmaz, O. T. , Toydemir, T. S. , Kirsan, I. , Dokuzeylul, B. , Gunay, Z. , & Karacam, E. (2014). Effects of surgical wound infiltration with bupivacaine on postoperative analgesia in cats undergoing bilateral mastectomy. Journal of Veterinary Medical Science, 76(12), 1595–1601. 10.1292/jvms.14-0112 25649941 PMC4300374

